# Effect of environmental enrichment devices on behavior of individually housed beef heifers

**DOI:** 10.1093/tas/txaa220

**Published:** 2020-11-28

**Authors:** Kelsey Bruno, Elizabeth DeSocio, Jason White, Blake K Wilson

**Affiliations:** Oklahoma State University, Department of Animal and Food Sciences, Stillwater, OK

**Keywords:** cattle, rope, rumination, scratch

## Abstract

In research settings, certain experimental designs may require cattle to be housed individually. Individual housing of cattle may make the animals more susceptible to boredom and result the development of undesirable behaviors. The objective of this trial was to investigate the effects of different environmental enrichment devices (EED) on the behavior and feed intake of heifers. Twenty mixed-breed single-sourced heifers were used in a completely random design. Heifers were housed individually (3.05 m × 3.66 m) with the ability to have physical contact with adjacent heifers. Heifers were randomly assigned to one of the four EED treatments, including a jolly ball (JLY), a broom head (SRCH), a 182 cm 5-knot rope (RP), or a Pas-a-Fier roller (RLR). Behavior was recorded using 8 h long daily instantaneous scan sampling in 30 min intervals over three periods: 7 d prior to EED addition (PR), 7 d with EED (EDP), and 7 d after removing EED (PST). Standing, laying, eating, drinking, and exploratory behaviors were evaluated. Exploratory behaviors included: interaction with water trough, feed bunk, water pipe, pen gate, pen wall, EED, grooming, or allogrooming. Rumination behavior was also recorded during each observation time. Time standing and standing bouts were greatest for RP (*P* < 0.05), while JLY and RLR spent the most time lying down (*P* < 0.05). All heifers spent the majority of observation times lying down, followed by solely standing (*P* < 0.05). Heifers on the RP treatment interacted the most with their EED, followed by SRCH (*P* < 0.001). Rumination increased during EDP compared to PR (*P* < 0.001). These results suggest that a RP suspended from the ceiling in the pen may be used most frequently by individually housed beef heifers.

## INTRODUCTION

Beef cattle are typically raised outdoors in an environment that offers variability however, some production settings require animals to be housed indoors in more barren environments. Wood-Gush and Beilharz (1983) suggested that long periods in a barren environment can lead to boredom, stress, and the development of stereotypies (repetitive behaviors that may be performed to cope with environment; Ridge et al., 2020). Some research settings may require beef cattle to be penned individually, either in small pens or stanchions, due to experimental design, such as for immune challenge experiments or intensive metabolic studies (Finck et al., 2014; Haque et al., 2017). During such trials, cattle may be isolated from conspecifics or limited to a small, barren environment, where grooming behaviors may be limited (McConnachie et al., 2018). Together, these conditions may make animals especially vulnerable to boredom, stress, and development of undesirable behaviors (Rushen et al., 1999). Additionally, social isolation may affect an animal’s ability to respond to novelty in its environment, respond to human stressors, and restraint (Kilgour et al., 2006).

Previous studies investigating environmental enrichment devices (EED) have found decreased negative social behaviors and reduced stress (Keeling et al., 2016; Park et al., 2020). Environmental enrichment devices typically include interactive objects that promote natural behaviors, reduce negative effects, and improve biological functioning (Keeling et al., 2016; Zobel et al., 2017). However, since the same EED have not been used consistently in previous work, results have varied depending on the EED used. Previous research with cattle has commonly used a scratching device (Wilson et al., 2002; Horvath et al., 2019; Park et al., 2020) or a manila rope (Zobel et al., 2017). Scratching devices may help to meet the animal’s natural grooming behavioral needs, scratch hard to reach places (DeVries et al., 2007), and increased grooming behavior may indicate a positive welfare state (Horvath et al., 2019). Objects that specifically elicit more oral involvement may be worth investigating because of their possible effect on non-nutritive oral behavior, even though the rope and brush may also stimulate oral behaviors.

Tongue-playing behavior, which involves moving the tongue from side to side outside the mouth, is a common stereotypy that results from cattle being under-stimulated (Ishiwata et al., 2008), or lack of forage availability (Ridge et al., 2020). Because this is a common outcome of a barren environment (Binev, 2020), EED that require tongue involvement may be more effective in stimulating cattle. Because cattle naturally often participate in social grooming (Val-laillet et al., 2009), these EED may also be more effective in an environment with limited social interaction, where additional grooming opportunities may replace social grooming.

The objective of this trial was to investigate which EED individually housed heifers would interact with most frequently and how EED affect overall pen exploratory behavior and feed intake.

## MATERIALS AND METHODS

### Ethical Approval of Animal Use

All animal procedures were approved by Oklahoma State University Institutional Animal Care and Use Committee (AG-15-6).

### Animals and Treatments

**Figure 1.**
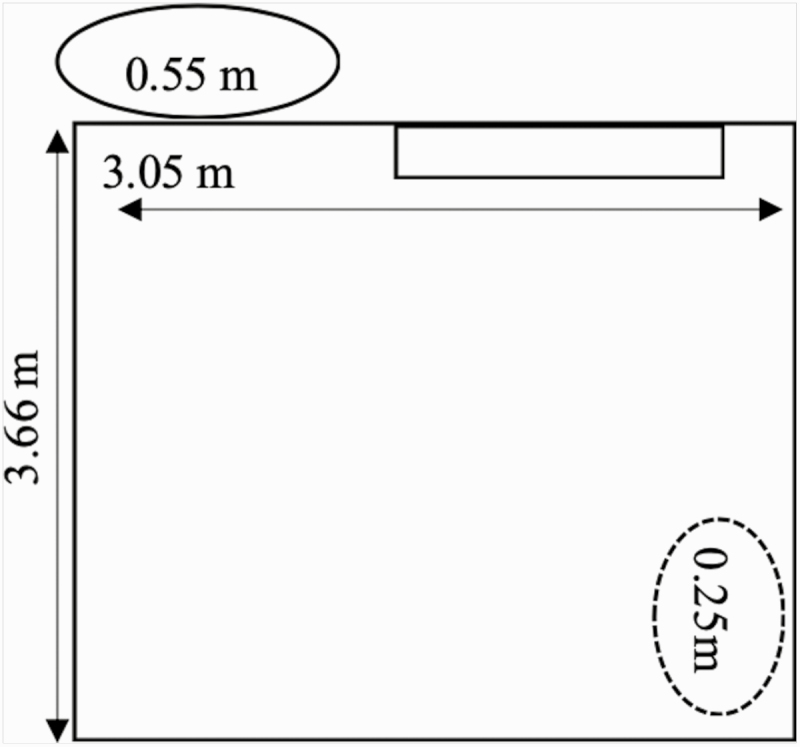
Picture of pen design outline. Solid lined oval is the feed bunk, 0.55 m wide. The pen has a 0.45 m × 1.27 m adjustable head lock leading to the feed bin. The dotted line oval represents a water bunk that is 0.25 m long and is in front of a 0.40 × 0.55 opening in the pen wall where heifers had contact with adjacent pens. The solid line rectangle represents the RLR or SRCH enrichment device mounted on the pen wall.

This trial was conducted at the Nutrition and Physiology Research Center (NPRC) at Oklahoma State University in Stillwater, OK, USA. Twenty mixed breed weaned beef heifers (BW = 292 ± 18 kg) were used in a completely randomized design for the 21-d experiment. Per routine processing procedures, upon arrival heifers were administered a prophylactic dose of tilmicosin phosphate (15 mg/kg BW, Micotil; Elanco Animal Health, Indianapolis, IN), doramectin (200 mg/kg BW, Dectomax; Zoetis, Florham Park, NJ), and vaccinated for viral and clostridial pathogens excluding BVDV (Inforce 3; Zoetis and Bar-Vac 7; Boehringer Ingelheim Vetmedica, St. Joseph, MO); heifers were ear tagged prior to arrival for individual identification. Heifers were maintained in individual slatted floor pens (3.05 m × 3.66 m; Figure 1) for 32 d prior to initiating the trial. Bedding was not provided in the pens as pens were cleaned by washing waste below the slatted floor, where the waste was removed through a drainage system.

Heifers were randomly assigned to individual pens. Each pen had two automatic water bowls and an individual feeding trough. Pens had vertical bars to allow for visual contact and the area of the pen with the water bowl had large enough holes in the wall such that heifers could have physical contact with the heifer in the neighboring pen (i.e., openings in pen wall above water troughs). Heifers were maintained in one area of the research facility and pens allowed for visual and tactile contact due to vertical bar walling with both adjacent pens. Pens were cleaned three times a week. Heifers were fed a ration (Table 1) formulated to meet or exceed all NASEM (2016) requirements once daily at approximately 0700 hours. Feed was managed using a modified “slick bunk management” system, allowing for minimal feed refusals. Feed refusals were collected daily and feed calls were adjusted accordingly. Ration samples were collected daily and DM analyses were performed. Heifers had free access to water at all times.

**Table 1.** Diet composition

Ingredient	Amount, % DM
SweetBran^*a*^	54.5
Prairie hay	30.0
Dry rolled corn	11.5
Supplement^*b*^	4.0
Analyzed composition	
NEm, Mcal/kg	1.73
NEg, Mcal/kg	1.11
TDN, %	73.42
CP, %	17.01
Crude fiber, %	14.93
ADF, %	19.34
NDF, %	41.86
Fat, %	3.05

^*a*^SweetBran, manufactured by Cargill, Dalhart, TX.

^*b*^Supplement contained Vitamin A 30,000 IU/g, Vitamin E 500 IU/g, Vitamin D 30,000 IU/g, Rumensin-90, and Tylan-40.

Heifers were examined daily for signs of disease. In order for a heifer to qualify for antimicrobial treatment, the heifer must have displayed clinical signs (e.g., lethargy, coughing, emaciation, etc.) and had a rectal temperature of 40 °C. No heifers met the criteria to receive antimicrobial treatment during this trial.

Heifers were randomly assigned to one of the four EED treatments (five heifers/treatment) using average mean differences and standard deviations similar to that by Wilson et al. (2002). A two-tailed power analysis with an alpha of 0.05 of five animals resulted in 0.93; thus, five animals were allotted per treatment. Environmental enrichment devices included: Jolly Tug-n-Toss Ball (JLY; Jolly Pets, Streetsboro, OH), a broom head (SRCH; Quickie, Cinnaminson, NJ), a 72-inch 5-knot rope tug (RP; Mammoth Pet Products, Mammoth Lakes, CA), and a Horse Pas-a-Fier (RLR; Horseman’s Pride Inc., Streetsboro, OH). The JLY is a large rubber ball with a handle allowing for the animal to grab the ball with the handle using the mouth to manipulate the EED. The RLR is a toy with raised nubs and a spinning center wheel that allowed for gum massaging and spinning with the lips and tongue. All EED were predominantly red to avoid bias due to color. Jolly Balls were left on the pen floor for free use, SRCH were attached to the gate of the pen at animal shoulder height (approximately 1.2 m), RP were suspended from the ceiling in the center of the pen on a spring, and RLR were attached to the gate of the pen at animal shoulder height. Enrichment devices were assigned at random, and the cattle in adjacent pens could see the EED in the pens on either side, but not access the EED in adjacent pens.

Behavioral observations were measured for 21 d, broken into three periods of 7 d: pre-enrichment device period (PR; 7 d prior EED; d 1–7), enrichment device period (EDP; 7 d with EED; d 8–14) and post-enrichment device period (PST; 7 d following removal of EED; d 15–21). During EDP, the EED were placed in the respective pens at 1900 hours on d 7; data collection for EDP began at 1000 hours on d 8. Enrichment devices were removed from pens at 1900 hours on d 14.

### Behavior Analysis

Observations were collected from 1000 to 1800 hours, daily. Prestudy observations indicated that heifers typically started eating after feed delivery and continued for approximately 2 to 2.5 h postfeeding. For that reason, observation times were initiated when eating behavior was less continuous (1000 hours) to avoid constant “eating” behavior measurements. Behavioral observation ended at 1800 hours because the lights in the NPRC were typically turned off at 1800 hours for the night. Heifers did not have access to natural light and were maintained on a 12:12 light:dark cycle starting at 0600 hours. This protocol was not altered to avoid disrupting conditioned behavior.

Heifers were visually observed every 30 min for 8 h/d for 21 d by one of two trained observers. Observers used an instantaneous scan sampling (Mitlohner et al., 2001) observation method in 30-min intervals such that each observer walked down the alley in between the pens and recorded the behavior of each heifer at that instant. One observer trained the other observer. Additionally, “getting up” was initially included in the observations to account for animals that may change lying behavior. The measure was removed from the analyses due to such infrequent occurrences. Thus, it was assumed the behavior of the heifers was not altered by observer presence. Possible behaviors exhibited by each heifer are listed in Table 2. If the heifer was participating in an “exploratory” behavior the location of that behavior was also recorded. The locations of exploratory behavior are also listed in Table 2.

**Table 2.** Ethogram of behaviors observed and whether or not location was recorded as well as definitions of possible locations of exploratory behavior

Behavior	Definition	Location recorded?
Drinking	Head in or over the water trough	No
Eating	Head in or over the feed bunk	No
Lying	Body contact with the ground	No
Standing	Upright posture, no locomotion, no interaction with other parts of pen,	No
Getting up	Heifer was originally observed in “lying” position, but as observed moved to the “standing” position	No
Exploratory	Interaction with a specific part of the pen or another heifer by heifer’s head, tongue, or body	Yes
Location	Definition	
Water trough	Heifer’s head, tongue, or body is actively touching the water trough. Heifer’s head is not in or over the water trough	
Feed bunk	Heifer’s head, tongue, or body is actively touching the feed bunk. Heifer’s head is not in or over the feed bunk	
Water pipe	Heifer’s head, tongue, or body is actively touching the water pipe	
Pen gate	Heifer’s head, tongue, or body is actively touching the pen gate	
Pen wall	Heifer’s head, tongue, or body is actively touching the pen wall. This does not include the pen gate	
Environmental enrichment device	Heifer’s head, tongue, or body is in contact with the enrichment device	
Grooming	Heifer is actively cleaning or licking itself	
Allogrooming	Heifer is actively cleaning or licking another heifer	

In addition to the listed behaviors, tongue behavior was also observed. Because two of the EED required mouth based interaction, it was hypothesized that tongue play may be affected. Therefore, at each observation, location of behavior and whether or not the heifer was tongue rolling or ruminating was recorded for each heifer. Rumination was defined as mastication movements other than eating. Dry matter intake (DMI) was measured each d by multiplying the “as fed” daily feed intake by the daily dry matter percentage. Daily DMI change was measured by subtracting the second d DMI from the first to look at daily change in intake.

Lastly, a HOBO Pendant G Data Logger accelerometer (Onset; Bourne, MA) was attached to the medial side of the right hind leg below the hock and above the metatarsophalangeal joint of each heifer to monitor lying and standing behavior 24 h a day. Loggers were attached to heifers at 1800 hours on d 0 and set to start recording at 0000 hours on d 1. Loggers were wrapped in cotton around each heifer’s leg, and then wrapped with vetrap (3M; Maplewood, MN) to hold loggers in place. Loggers were programmed to record in 1-min intervals with the x-axis pointed toward the cranial direction and the y-axis pointed toward dorsal direction. Accelerometers were downloaded on d 22 after being removed from each heifer using Onset HOBOware Software.

### Statistical Analysis

Behaviors were monitored for each heifer each day. Behavior frequency was summed for each behavior of each animal for each day for analyses. The response variable was frequency of behavioral occurrences, or count, for each animal on each day. Behavior data were analyzed using the GLIMMIX procedure of SAS version 9.4 (SAS Institute Inc.; Cary, NC) allowing for repeated measures. The model statement included treatment and day. Day was included as a repeated measure. Data were also analyzed by period (as opposed to day) to compare the three periods (PR, EDP, and PST); this method was analyzed the same way, replacing day with period. Locations of behaviors and rumination behavior were analyzed using the same method. The “getting up” behavior and “drinking” behavior happened too infrequently to be statistically analyzed and thus were removed from analysis.

Locomotor behavior, DMI, and daily DMI change were analyzed using the GLIMMIX procedure of SAS. Accelerometer data was divided into standing time, standing bouts, standing duration, lying time, lying bouts, and lying duration. The model included effects of treatment, period, and the interaction. One accelerometer was lost during the last week of the trial and as a result, accelerometer data for that heifer was lost. Thus, there are four heifers represented for RP treatment rather than five for locomotor behavior.

Main effects were considered significant when *P* ≤ 0.05 and a trend was considered when 0.05 < *P* ≤ 0.10.

## RESULTS AND DISCUSSION

### Environmental Enrichment Device Interaction

**Figure 2.**
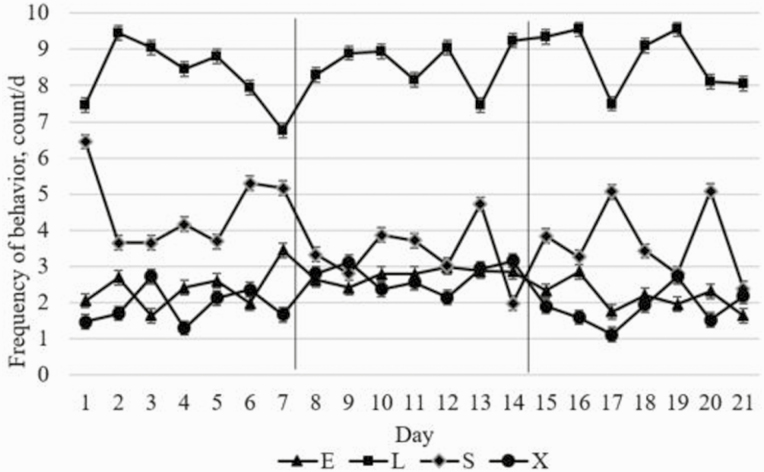
Day effect for 21-day trial period on frequency of each behavior observed for each day. Behaviors are abbreviated as E for eating, L for lying, S for standing, and X for exploratory. Lines on graph refer to study periods^.^ The three periods of 7 d: pre-enrichment device period (PR), enrichment device period (EDP) and post-enrichment device period (PST).

There was an interobserver reliability (joint probability of agreement) of 95% between the two observers. There were no treatment by day interactions for any behaviors (*P* ≥ 0.28). There was no effect of treatment on behavior (*P* ≥ 0.22), except for exploratory behavior during d 8 to d 14, where RP heifers spent more time performing exploratory behavior compared to other treatments (*P* = 0.05). There was a significant day effect for all days of the trial (*P* ≤ 0.05; Figure 2) such that heifers were more frequently observed lying each day compared to other behaviors. The disproportional amount of time that all heifers spent lying compared to other behaviors was not surprising, as they were in a confined space allowing for limited movement. Feedlot cattle in confinement have also been found to spend more time lying than standing (Mitlohner et al., 2001; MacKay et al., 2013). It is noteworthy that standing time numerically decreased the day that EED were placed in the pens, possibly indicating that heifers may have been more uncomfortable or restless while EED were being assembled in pens, assuming that comfortable or resting heifers would spend more time laying down in their pens.

**Figure 3.**
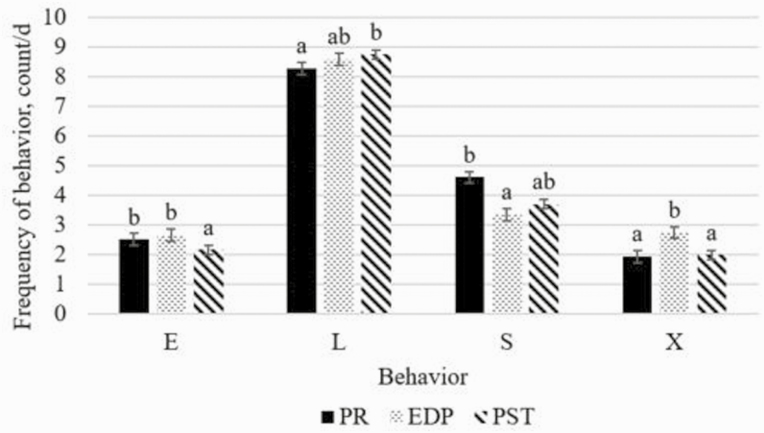
Effects of experimental period on pen behaviors. Behaviors are abbreviated E for eating, L for lying, S for standing, and X for exploratory. PR was d 1 to d 7, EDP was d 8 to d 14, PST was d 15 to d 21. ^ab^Indicates significant differences (*P* ≤ 0.05).

**Figure 4.**
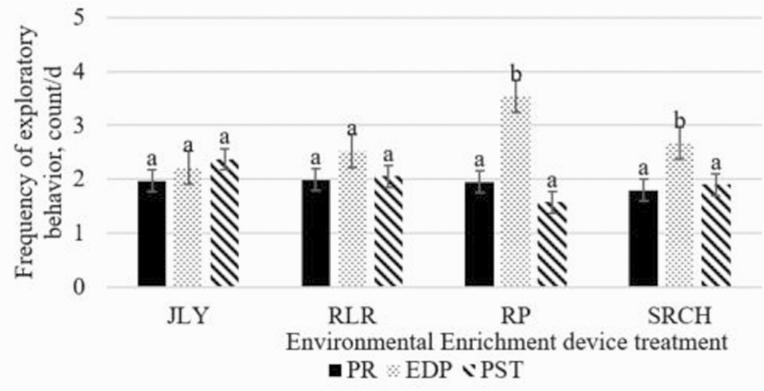
Effects of period by EED treatment interaction on exploraotry behavior. Environmental enrichment devices included: Jolly Tug-n-Toss ball (JLY; Jolly pets, Streetsboro, OH), a broom head (SRCH; Quickie, Cinnaminson, NJ), a 72-inch 5-knot rope tug (RP; Mammoth pet products, Mammoth Lakes, CA), and a Horse Pas-a-Fier (RLR; Horseman’s Pride Inc., Streetsboro, OH). PR was d 1 to d 7, EDP was d 8 to d 14, PST was d 15 to d 21. ^ab^Indicates significant differences (*P* ≤ 0.05).

**Figure 5.**
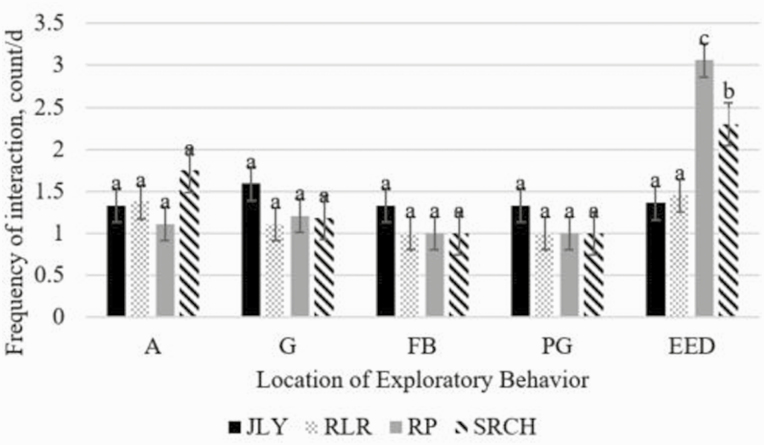
Effect of treatment on location of exploratory behaviors. Environmental enrichment devices included: Jolly Tug-n-Toss ball (JLY; Jolly pets, Streetsboro, OH), a broom head (SRCH; Quickie, Cinnaminson, NJ), a 72-inch 5-knot rope tug (RP; Mammoth pet products, Mammoth Lakes, CA), and a Horse Pas-a-Fier (RLR; Horseman’s Pride Inc., Streetsboro, OH). Locations are allogrooming (A), grooming (G), feed bunk (FB), pen gate (PG), and environmental enrichment device (EED). RP and SRCH heifers interacted with their enrichment device more frequently than other treatments (*P* ≤ 0.001). ^abc^Indicates significant differences (*P* ≤ 0.01).

When analyzed as periods, there was no period by treatment interaction for eating, lying, or standing (*P* ≥ 0.27), but there was for exploratory behavior, such that RP and SRCH heifers had increased exploratory behavior during EDP (*P* ≤ 0.001; Figure 4). When analyzed as periods, there was a period effect such that standing time decreased during EDP compared to PR, lying behavior increased from PR to PST, eating behavior decreased from PR and EDP to PST, and exploratory behavior increased during EDP compared to PR and PST (*P* ≤ 0.01; Figure 3). When analyzed by period, there was a treatment effect on location of exploratory behavior, where RP and SRCH heifers interacted with EED more frequently than other locations (*P* ≤ 0.001; Figure 5), but no difference was observed for A, G, FB, or PG. Interactions with pen wall, water pipe, and water trough happened too infrequently to be analyzed.

The increase in exploratory behavior when EED were introduced was expected. Increased environmental exploration is important when EED are introduced (Mench, 1998) and the increase was likely driven by the addition of the EED. Additionally, the daily frequency of exploratory behavior during EDP did not differ, indicating that habituation to EED did not take place. Wilson et al. (2002) found that there was no habituation with a scratching EED. Wilson et al. (2002) suspected that habituation would occur over a longer period of time, which might have happened in this trial if heifers had access to EED for more than 7 d. DeVries et al. (2007) stated that scratching EED would be best for reaching inaccessible places while grooming, like the back and hindquarters. Horvath et al. (2019) reported an increase in macro bout frequency of self-grooming behavior with the addition of a mechanical brush and no difference in allogrooming behaviors. While an increase in personal grooming was not observed in the individual animal, they may have used the device to access areas they cannot normally reach. Future studies should note parts of the body that contact the scratching EED. Additionally, Horvath et al. (2020) reported that intervals above 3 min while scan sampling for brush use were less reliable. Thus, the intervals chosen for this trial may have been too large to identify true sought differences in EED use.

The RP treatment was the most frequently explored EED by the heifers. The RP might have stimulated play behavior because it was hanging from a spring attached to the ceiling and had some movement or “give,” while the SRCH did not move. The RLR was also fixed to the pen gate, but the rolling part of the device did allow for some movement. The JLY was also a moveable device, but being on the floor was prone to collect dirt and feces, which may have discouraged interaction. Wilson et al. (2002) suggested that a movable scratching device stimulated play behavior because of the movement of the device. Because heifers could push, rub, or pull the rope it may have been the most “play friendly” EED, facilitating the most interaction. Stanford et al. (2009) offered one or two ropes in cattle pens and monitored interaction for 14 wk; the authors found continuous interaction with ropes and did not indicate habituation. Rope EED have also been used more frequently in other studies with piglets (Trickett et al., 2009). Trickett et al. (2009) suggested that RP may have been more popular compared to wood blocks due to the hanging presentation or because it was an object able to be manipulate the object’s integrity; Pelley et al. (1995) also made this observation about why a bale of straw was more popular than salt blocks and broom heads. The ability to truly manipulate may have also stimulated interest by the heifers; although, none of the ropes were noted as damaged after they were taken down.

Previous work has reported that SRCH EED were most frequently used, but SRCH was the second most used EED in this trial. Wilson et al. (2002) stated that heifers interacted more frequently with a scratching object than either of two scented objects, where the authors investigated two types of scratching objects and two scented objects. DeVries et al. (2007) stated that heifers spent less time interacting with other areas of the pen (pen wall and water trough) when offered a scratching brush. In this trial, there was no difference in frequency of interaction of other pen locations between the first and second period. The brush devices used by Wilson et al. (2002) and DeVries et al. (2007) were both larger than those used in the current trial and provided more opportunity for cattle to reach inaccessible places. Additionally, the two aforementioned trials utilized group housed calves, making the comparisons to this trial limited. The SRCH EED used in this study would be comparable to that used in Pelley et al. (1995), which was smaller and less mobile. Thus, SRCH EED may be more useful when they are larger and able to reach more inaccessible areas of the body. Yet, those larger SRCH objects are not easily implemented into smaller pen settings used in intensive research data collection, such as the pen setting in this study. The location and distance from the feed bunk of the device can also affect use (Mandel et al., 2015). Most devices in this trial were close to the feed bunk and mounted on the pen gate. Thus, this location might not have been ideal for usage and future work should alter location of mounted EED.

Mench et al. (1998) suggested that scratching devices may affect stereotyped behaviors such as tongue rolling and biting because the massaging effect of a scratching device may stimulate normal mouth movements in heifers. Park et al. (2020) reported that addition of brush EED increased tongue rolling bouts and duration in feedlot cattle. Similarly, Zobel et al. (2017) reported non-nutritive suckling being directed at the rope EED in their trial. While both EED have been reported to affect oral behaviors, this trial included EED that specifically involved tongue manipulation to test if those types of EED would be better suited to control stereotypies than a scratching device. Because no tongue rolling or tongue related stereotypies were observed in this study, it cannot be concluded which device would impact tongue rolling. Other authors have suggested that tongue rolling is more related to dietary effects, specifically low fiber. Faleiro et al. (2011) found that frequency of stereotypies were decreased in cattle supplemented with barley straw and Iraira et al. (2013) found decreased frequency of tongue rolling with a higher fiber diet. Although the fiber content of the diet used in this trial is close to the content of their low fiber diet, it still did not amount to observations of tongue rolling.

Use of some EED may require settings that allow for social mimicry. Stanford et al. (2009) suggested that interaction may be related to the ability to observe penmates interacting with objects. This may have affected interaction with the RLR, because it requires significant curiosity to fully interact and heifers were housed individually. A pen setting may allow for more curious animals to lead EED interactions. All studies previously mentioned (Pelley et al., 1995; Wilson et al., 2002; DeVries et al., 2007; Trickett et al., 2009; Park et al., 2020) have used animals housed in a social pen setting, whereas this study housed heifers individually, but with the ability to have contact with neighboring heifers. Although heifers are not commonly housed individually, it seems that use of a simpler EED should be used in these pen settings during intensive research data collection.

### Accelerometer Behavior

Treatment significantly affected standing time, duration of standing bouts, and number of standing bouts such that standing time and number of standing bouts were greatest for RP heifers and least for JLY heifers (*P* ≤ 0.01; Table 3), while JLY heifers had the longest standing bout durations (*P* ≤ 0.05; Table 4). Overall, JLY heifers spent the most time lying (*P* ≤ 0.01). Average duration of lying bouts decreased from PR to PST, while overall number of lying bouts increased from PR to EDP (*P* ≤ 0.01; Table 5).

**Table 3.** Main effects of environmental^*a*^ enrichment device treatment on standing and lying behavior,^*b*^ dry matter intake, and ruminating frequency

	JLY	RLR	RP	SRCH	SEM	*P*-value
Standing						
Time,^*c*^ min/d	539^c^	565^b^	616^a^	572^b^	10.8	<0.01
Average duration of bouts,^*d*^ min/d	84^a^	68^b^	77^ab^	72^b^	4.7	0.04
Bouts,^*e*^ number/d	7.9^b^	8.8^a^	9.1^a^	8.3^ab^	9.05	0.02
Lying						
Time^*c*^, min/d	900^a^	874^ab^	846^b^	867^b^	14.7	0.04
Average duration of bouts,^*d*^ min/d	36	32	38	34	2.9	0.47
Bouts, number/d	36	35	36	33	2.3	0.64
DMI, kg	16.8^a^	14.0^b^	12.8^c^	14.1^b^	0.33	<0.01
DMI change, kg	−0.02	0.15	0.07	0.11	0.138	0.85
Ruminating, count/d	8.7	8.6	8.6	8.5	0.17	0.94

^a,b,c^ Values with differing superscripts differ (*P* ≤ 0.05).

^*a*^Environmental enrichment devices included: Jolly Tug-n-Toss ball (JLY; Jolly pets, Streetsboro, OH), a broom head (SRCH; Quickie, Cinnaminson, NJ), a 72-inch 5-knot rope tug (RP; Mammoth pet products, Mammoth Lakes, CA), and a Horse Pas-a-Fier (RLR; Horseman’s Pride Inc., Streetsboro, OH).

^*b*^Behavior measurements were collected using Onset Pendent G data loggers (Onset Computer Corp., Bourne, MA).

^*c*^Total duration of standing or lying per heifer.

^*d*^Time of continuous lying or standing behavior per heifer within a bout.

^*e*^Number of changes between standing to lying or lying to standing per heifer.

**Table 4.** Main effects of period^*a*^ on standing and lying behavior,^*b*^ dry matter intake, and ruminating frequency

	PR	EDP	PST	SEM	*P*-value
Standing					
Time^*c*^, min/d	576	569	574	8.6	0.74
Duration of bout,^*d*^ min/d	74	77	76	3.7	0.86
Bouts,^*e*^ number/d	8.6	8.6	8.5	0.23	0.94
Lying					
Time,^*c*^ min/d	881	872	863	10.6	0.80
Average duration of bouts,^*d*^ min/d	39	34	32	2.4	0.11
Bouts,^*e*^ number/d	31^a^	37^b^	37^b^	1.8	0.02
DMI, kg	14.1	14.2	14.9	0.3	0.13
DMI change, kg	-0.01	0.13	0.11	0.107	0.63
Ruminating, count/d	8.3^b^	9.1^a^	8.5^b^	0.15	< 0.01

^a,b,c^ Values with differing superscripts significantly differ (*P* ≤ 0.05).

^*a*^Three periods of 7 days: pre-enrichment device period (PR), enrichment device period (EDP), and postenrichment device period (PST).

^*b*^Behavior measurements were collected using Onset Pendent G data loggers (Onset Computer Corp., Bourne, MA).

^*c*^Total duration of standing or lying per heifer.

^*d*^Time of continuous lying or standing behavior per heifer within a bout.

^*e*^Number of changes between standing to lying or lying to standing per heifer.

Accelerometer measured standing time was greatest for RP heifers, which has been seen previously. Pelley et al. (1995) found that steers with a suspended EED spent the most time standing and ruminating compared to animals with other EED. The heifers might have spent more time standing to interact more with the RP. They also had longer standing durations and more frequent standing bouts, which could relate to time spent exploring the EED. Standing time was also high in the SRCH heifers, which again may be due to time spent exploring the enrichment device. Park et al. (2020) did not find a difference in standing and lying time between cattle with or without a mechanical brush. However, their data was collected using a scan sampling method and cattle were housed in pens. The continuous measuring of the accelerometers allowed for more sensitive differences in standing and lying to be detected.

Heifers with the RLR had frequent but shorter duration standing bouts, indicating more “getting up and down” behavior. This behavior can be indicative of discomfort or restlessness (Huzzey et al., 2005). Thus, the RLR may not have positively affected pen behavior of individually housed heifers. Since JLY was the only EED that could be interacted with in the lying position, it may have allowed for more lying time, whereas heifers had to be standing to explore other devices. Time standing may affect hoof health and increased time standing could lead to increased lameness (Galindo and Broom, 2000). Although no differences in hoof health were observed in this trial, the length of observation may have been too short for such conditions to develop. Although no differences in hoof health were observed, there may have been differences in comfort still that could lead to differences in performance.

### Dry Matter Intake

There was no interaction between treatment and period for DMI. There was a significant effect of treatment on DMI, where JLY heifers had the highest overall DMI (*P* ≤ 0.01), but there was not a period effect on DMI (*P* = 0.53). Daily change in DMI was not different between EED or periods (*P* ≥ 0.51). These results indicate that the EED treatments did not affect DMI. Similarly, Trickett et al. (2009) found no difference in DMI in piglets that were given EED.

There was no difference in rumination between treatments (*P* ≥ 0.12; Table 3). There was a significant day effect on rumination for both PR and PST (*P* ≤ 0.01; Table 5) where rumination was variable each day. Ruminating behavior increased during EDP (*P* ≤ 0.001), but was not different between EED treatments. Pelley et al. (1995) observed increased ruminating behavior in animals offered a SRCH device. However, Ishiwata et al. (2006) observed decreased ruminating behavior when steers were enriched with a drum can device. Similarly to the results of this trial, Park et al. (2020) also found no difference in rumination duration in feedlot cattle with a brush enrichment device. This suggests that the effect of EED on ruminating may depend on the EED. Differences may be specific to pen condition and devices used.

The results of this trial highlight some considerations for the inclusion of EED to cattle being housed in barren pens individually. These results show that RP based EED may be used most frequently in these pen settings, especially if suspended and allowing for maximal manipulation, compared to the other EED evaluated. While the SRCH EED was not used the most in this study, a larger or mobile scratching device may be utilized more frequently in a larger, social pen setting. Thus, when selecting an EED, one must consider pen and social conditions of housing to determine the optimal device. Future similar studies should consider physiological differences associated with welfare and each EED when investigating differences between individually housed beef heifers.
